# Dexpramipexole Is Ineffective in Two Models of ALS Related Neurodegeneration

**DOI:** 10.1371/journal.pone.0091608

**Published:** 2014-12-19

**Authors:** Fernando G. Vieira, Eva LaDow, Andy Moreno, Joshua D. Kidd, Beth Levine, Kenneth Thompson, Alan Gill, Steven Finkbeiner, Steven Perrin

**Affiliations:** 1 ALS Therapy Development Institute, Cambridge, Massachusetts, United States of America; 2 Gladstone Institute of Neurological Disease, San Francisco, California, United States of America; 3 Taube-Koret Center for Neurodegenerative Disease Research and the Hellman Family Foundation Alzheimer's Disease Research Program, San Francisco, California, United States of America; 4 Departments of Neurology and Physiology, University of California San Francisco, San Francisco, California, United States of America; University of Sheffield, United Kingdom

## Abstract

Treatment options for people living with amyotrophic lateral sclerosis (ALS) are limited and ineffective. Recently, dexpramipexole (RPPX) was advanced into human ALS clinical trials. In the current studies, we investigated RPPX in two parallel screening systems: 1) appropriately powered, sibling-matched, gender-balanced survival efficacy screening in high-copy B6-SJL-SOD1^G93A^/Gur1 mice, and 2) high-content neuronal survival screening in primary rat cortical neurons transfected with wild-type human TDP43 or mutant human TDP43. In both cases, we exposed the test systems to RPPX levels approximating those achieved in human Phase II clinical investigations. In SOD1^G93A^ mice, no effect was observed on neuromotor disease progression or survival. In primary cortical neurons transfected with either mutant or wild-type human TDP43, a marginally significant improvement in a single indicator of neuronal survival was observed, and only at the 10 µM RPPX treatment. These systems reflect both mutant SOD1- and TDP43-mediated forms of neurodegeneration. The systems also reflect both complex non-cell autonomous and neuronal cell autonomous disease mechanisms. The results of these experiments, taken in context with results produced by other molecules tested in both screening systems, do not argue positively for further study of RPPX in ALS.

## Introduction

Amyotrophic lateral sclerosis (ALS) is a progressive neurodegenerative disease defined by upper and lower motor neuron failure. [1] Treatment options exist for the management of symptoms and complications relating to ALS, but only riluzole treatment has been approved by regulatory agencies to slow ALS disease progression. [2]–[3] Riluzole's efficacy is marginal. Indeed, repeated clinical studies have demonstrated only limited improvements in survival, without benefits in motor function. [2] Efforts to uncover better treatments to slow, stop, or reverse neurodegeneration in ALS continue.

In the past two decades, important advances have been made in identifying genes that predispose individuals to developing inherited ALS. Superoxide dismutase 1 (SOD1) was the first gene to be identified as causing ALS when mutated. [4] Cell-based and animal models feature impaired SOD1 function or over-express wild-type or mutated SOD1. The best characterized is the high-copy B6-SJL-SOD1^G93A^/Gur1 mouse, a heterozygous transgenic mouse that ubiquitously expresses more than 20 copies of mutant human SOD1. [5]–[6] It recapitulates many of the pathological hallmarks of both human familial and sporadic ALS, including muscle weakness, atrophy, motor neuron death, protein aggregation, and more. These high-copy B6-SJL-SOD1^G93A^/Gur1 mice have been used to better understand ALS pathogenesis and also to screen and test potential therapeutics to justify a human ALS clinical trial.

In 2008, after identification of neuronal cytoplasmic 43 kDa Tar DNA binding protein (TDP43) as a prominent pathological hallmark in both familial and sporadic ALS, mutations in the gene were identified in familial cases of ALS. [7]–[8] Studies have implicated RNA metabolism in ALS disease pathogenesis that may be independent from SOD1-mediated ALS. [9] While no transgenic TDP43 rodent model has been identified yet which consistently demonstrates a phenotype suggestive of human ALS[10], cell-based systems have been developed which model some elements of TDP43 neuronal pathology. [11]–[12] For example, the TD43 model used in the study that follows recapitulates a number of features seen in ALS patients including an increased propensity of mutant TDP43 to mislocalize to the cytoplasm, to aggregate into detergent resistant inclusion bodies, to induce the loss of neurites and to lead to accelerated neuronal death. These models, when coupled to high-content screening technologies, can be valuable for elucidating whether a drug might be protective against TDP43 mediated cytotoxicity. [13]

Despite these advances in our understanding of ALS and the development of therapeutic screening models, no single preclinical assay or a set of assays can be unequivocally considered to be “gate-keepers” for clinical testing in ALS. [14] Further, the only positive control pharmacological agent against which to benchmark clinical therapeutic candidates for ALS is riluzole. [14] With its marginal clinical and preclinical efficacy, selection and prioritization of clinical candidates with similar preclinical efficacy profiles to that of riluzole is difficult.

Recently, dexpramipexole (RPPX) was advanced into human ALS clinical trials without the benefit of published, rigorous preclinical testing in models relating to either SOD1 or RNA binding protein–mediated neurodegeneration. The published work testing RPPX in high-copy C57-B6-SOD1^G93A^/Gur1 by Danzeisan and colleagues was completed prior to establishment of consensus guidelines for preclinical testing in animal models of ALS. The survival efficacy studies reported used underpowered cohorts and were not gender balanced. We now understand that these study design limitations can confound interpretation of results. [15] Notwithstanding the dearth of published data demonstrating efficacy in ALS related preclinical models, the molecule was advanced through three phases of human clinical testing in ALS patients. While it is unclear what ALS related preclinical studies Knopp Pharmaceuticals and BiogenIdec completed or had access to prior to advancing through their human ALS clinical studies, it is clear to the field that RPPX was well mostly tolerated in humans and demonstrated favorable human pharmacokinetics for central nervous system indications. [16] These attributes may have played a role in the decisions to move forward with the drug through clinical Phase III testing in ALS.

RPPX is the pure R+ enantiomer of pramipexole, a non-ergot dopamine agonist that is approved for treatment of Parkinson disease and restless leg syndrome. It has binding affinity for D2, D3, and D4 dopamine receptors. [16] Reports suggest RPPX is generally neuroprotective, and inhibits the opening of the mitochondrial permeability transition pore (PTP) *in vitro* and *in vivo*. [17]–[19] In 2003, a small clinical study demonstrated that serum from patients with sporadic ALS had elevated levels of a marker of free radical activity. The marker was reduced by treatment with pramipexole. [20] Studies directly comparing pramipexole to RPPX suggested that neuroprotection, mitochondrial PTP modulation, and oxygen species scavenging were independent of dopamine receptor binding. This was inferred because RPPX bound poorly to dopamine receptors yet retained neuroprotective effects. [20] Because it is a poor dopamine receptor agonist, RPPX has a greater neuroprotective therapeutic index than pramipexole. These characteristics were thought to make it a reasonable candidate for mechanistic, preclinical, and clinical studies in ALS. [17], [19], [21], [22]

Phase I and Phase II clinical trials studying RPPX in people living with ALS demonstrated that it was well tolerated at the dose levels tested. These trials also demonstrated trends toward dose-dependent efficacy against secondary endpoints tracking disease progression. [18], [23] A joint rank methodology integrating mortality and ALS-FRS score into a single ranking metric, provided preliminary evidence for a clinically significant benefit by RPPX, when compared to placebo control. Most recently, results from a large Phase III clinical trial that enrolled 943 persons living with ALS testing for efficacy of RPPX failed to demonstrate significant benefit when compared with placebo control. [16] Notably, only one small study of RPPX in a preclinical animal model of ALS has been published. In that report, RPPX was efficacious in B6-SJL-SOD1^G93A^/Gur1 mice, extending survival by approximately 10 days. [24]

In the current studies, we sought to investigate RPPX in two parallel screening systems: 1) appropriately powered, sibling-matched, gender-balanced survival efficacy screening in high-copy B6-SJL-SOD1^G93A^/Gur1 mice, and 2) high-content neuronal survival screening in primary rat cortical neurons transfected with wild-type human TDP43 or mutant human TDP43. In both cases, we aimed to expose the test systems to drug levels approximating those achieved and reported in human Phase II clinical investigations. [21]

## Results

### B6-SJL-SOD1^G93A^/Gur1 mouse survival efficacy study design and results

Mice chronically consumed dexpramipexole dihydrochloride dissolved in drinking water, at a concentration designed to deliver steady-state plasma levels of about 700 ng/mL. This concentration was based on dose-delivery verification studies that tested plasma levels during chronic treatment in drinking water. Dosing started at 55 days of age and continued until death. B6-SJL-SOD1^G93A^/Gur1 mice were assigned to two cohorts that were gender-balanced and litter-matched. *Ad libitum* administration of enantiomerically pure RPPX in drinking water started at 55 days of age and continued until death. All 32/32 drug group animals, and 32/32 vehicle control group animals, completed the study. Three females in the control group and one female in the drug group were right-censored in time-to-event analyses because they were still alive at age 180 days, the time of study termination. These mice expressed less SOD1 mRNA than typical B6-SJL-SOD1^G93A^/Gur1 mice in our colony, thereby resulting in extended survival (data not shown). [25]

#### Formulation Stability Studies

PPX is known to be relatively orally bioavailable. For these studies, we sought to deliver RPPX orally in drinking water. Prior to the initiation of any in vivo or cell based studies, we completed studies to determine whether and for how long RPPX would be stable in a drinking water with a 1 week duration being the upper time limit tested. We learned that there was no detectable degradation of RPPX in drinking water over seven days (Table 1, S1a-b Fig.).

**Table 1:** Dexpramipexole Stability in Drinking Water over One Week by HPLC.

Sample Solution	87.33 mg/mL RPPX solution	43.67 mg/mL RPPX solution	8.73 mg/mL RPPX solution
**Area % RPPX, Day 0**	95.4	94.7	95.0
**Area % RPPX, Day 7**	94.4	94.9	95.2

#### Dose-Delivery Verification Studies

**Figure 1:**
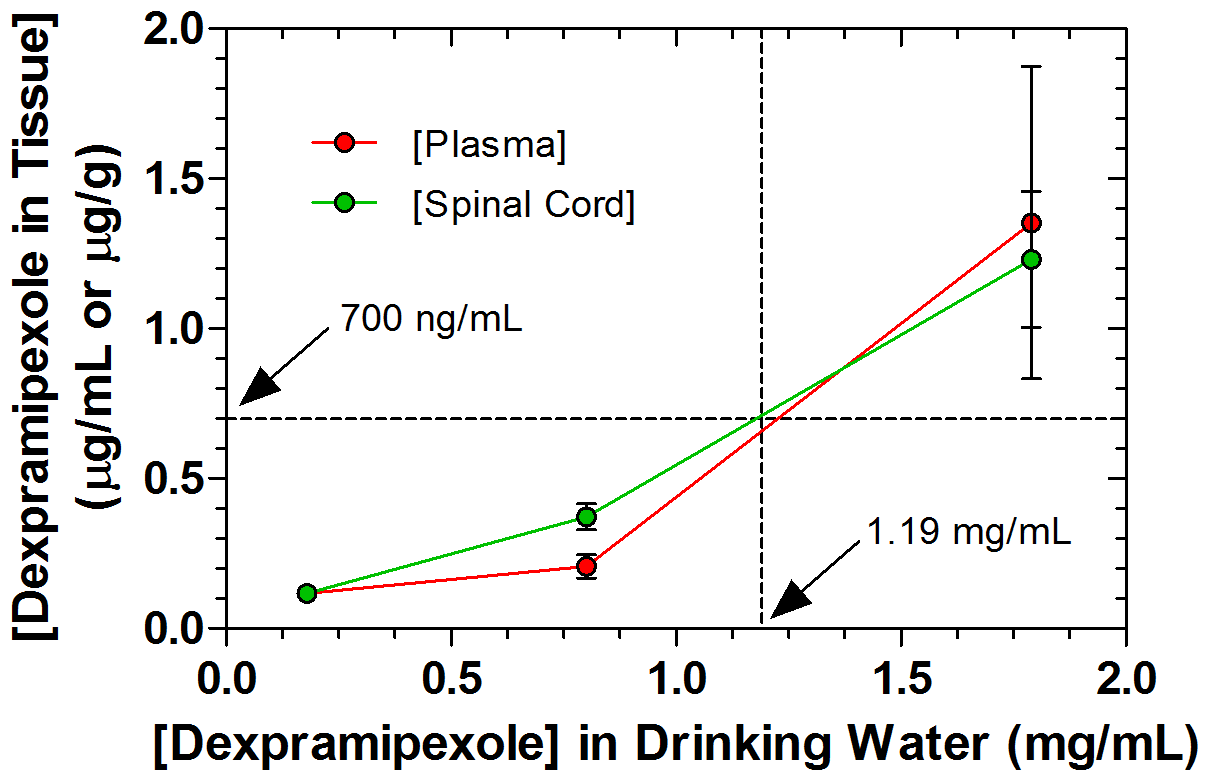
Steady-state dexpramipexole concentrations in SOD1^G93A^ mice receiving drug ad libitum in drinking water. Three males and three females in each group received 0.179, 0.9, or 1.79 mg/mL dexpramipexole dihydrochloride in the drinking water. Mean ± standard error of the mean concentration in plasma and spinal cord at steady-state after 14 days of treatment is shown at each dose level. A concentration of 1.19 mg/mL in the drinking water was chosen for the survival study in SOD1G93A mice to emulate steady-state concentrations attained by the 300 mg/d group reported in the human clinical trial (1).

We provided RPPX in the drinking water at 0.179, 0.9, or 1.79 mg/mL concentrations (providing 30, 150, or 300 mg/kg/d in 4.5 mL daily drinking volume) to separate groups of SOD1^G93A^ mice (3M, 3F per group) to determine plasma and spinal cord levels of RPPX at steady state after 14 days of treatment. Fig. 1 shows approximate steady state plasma and spinal cord concentrations after 14 days of treatment at each concentration in the drinking water. Because samples were harvested approximately eight hours after the start of the light cycle, it was assumed that RPPX levels observed might be closer to trough daily levels than to peak. From these data, a concentration of 1.19 mg/mL in the drinking water was chosen for our survival study in SOD1^G93A^ mice to roughly emulate steady-state concentrations attained by the 300 mg/d group in the human clinical trial. [23]

#### Body Weight

Failure to maintain body weight is an indicator of disease onset and progression in the SOD1^G93A^ mouse model of ALS. At age 55 days, the starting body weights averaged 20.5±0.63 and 20.6±0.69 g for the vehicle control and RPPX-treated groups, respectively (females 17.4±0.49 and 17.1±0.32; males 23.6±0.35 and 24.0±0.51).

**Figure 2:**
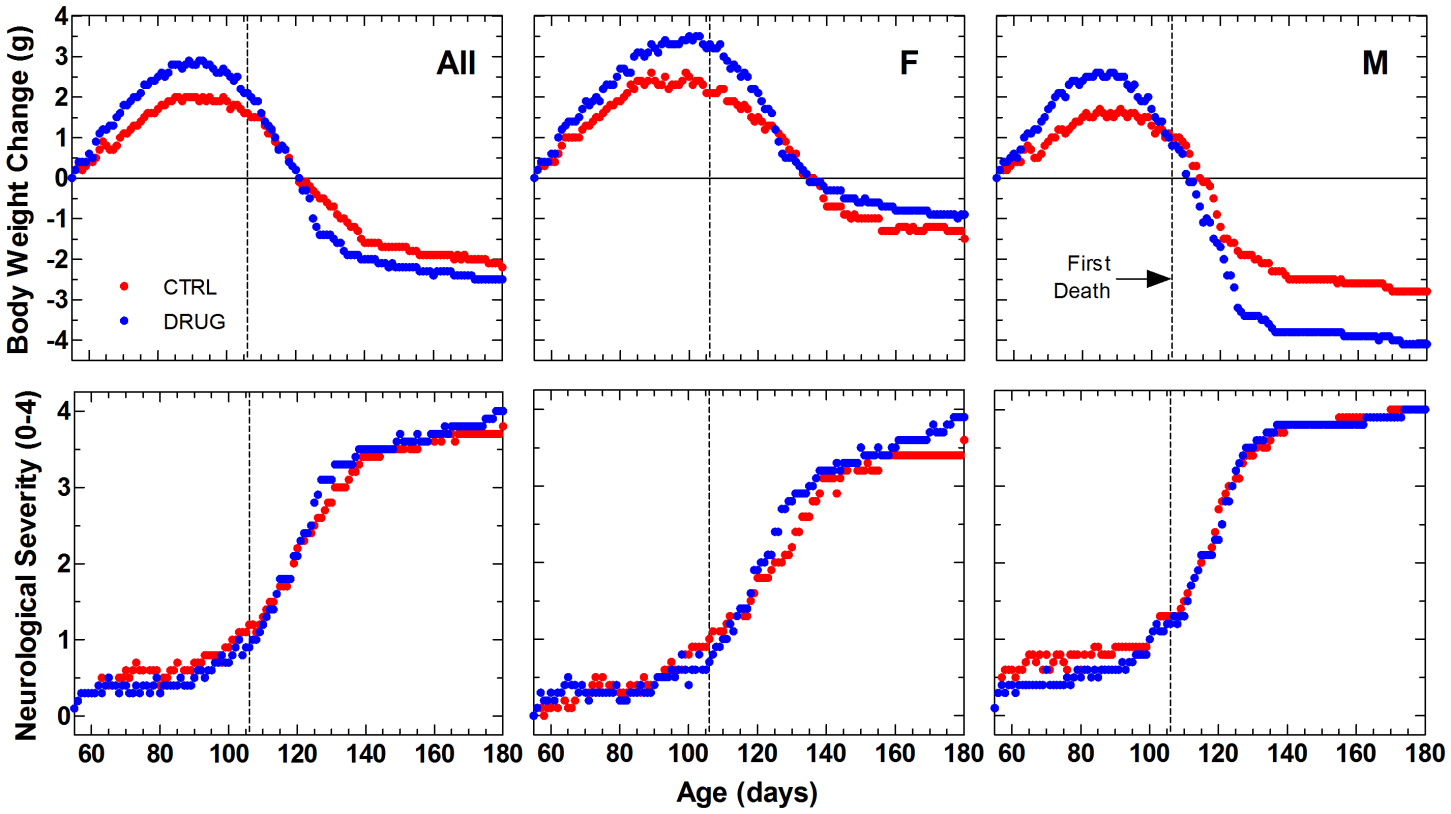
Daily average body weight change and neurological severity score in SOD1^G93A^ mice. Group average change from initial body weight (top panels) and neurological disease severity score (bottom panels) are compared over time from age at study start to age 180 days. Left panels show data from all animals (All); middle and right panels show data from females (F) and males (M), respectively. Vehicle control (CTRL) animals received 0.22 micron filtered animal facility drinking water. Dexpramipexole-treated (DRUG) animals received daily 200 mg/kg/day dexpramipexole (1.19 g/L) in filtered drinking water. Because animals died at different ages, group means were computed after carrying forward last values for each animal through 180 days of age. Results of statistical analysis for summary measures taken from these data are given in Table 2.

The daily average change in body weight of each treatment group was plotted over time for 180 days (study termination) (Fig. 2, top panels). To conserve the mean values as animals began to die, the last values were carried forward during computation of the means. Both male and female animals in the drug group showed a greater body weight gain (up to 0.9 g) during the ascent to peak body weight.

When examined separately for each animal, peak body weight occurred at different ages. When body weight change over time was fitted for each animal and measures derived from the individual curves, time to peak body weight and time from peak to terminal body weight were similar in both groups (Table 2). The average maximum (+0.6 g, p = 0.12) and median (+0.6 g, p = 0.07) increase from initial body weight tended to be greater in RPPX-treated animals (Student's 2-tailed independent t-test). Based on individual changes in body weight, chronic dosing with RPPX tended to show a potentially beneficial effect on disease onset or progression in these studies. These differences based on summary measures from individual animals' body weight changes were consistent with those shown by group average body weights over time. Longitudinal data analysis of body weight over time by mixed-effects maximum likelihood regression with random effects for mouse nested within random effects for litter, showed that RPPX-treated animals were, on average, about 0.5 g heavier than controls. [23] However, this effect was not statistically significant (p = 0.12). Thus, RPPX-treated animals showed a tendency to better maintain body weight over time than did control animals; however, this effect was not statistically significant.

**Table 2:** Change from Initial Body Weight over Time.^1^^,^ 2

Parameter	Measure	Subjects	Treatment	N	Mean	D-C	P
Time (days)	Initial to Peak	All	CTRL	32	45.4		
			DRUG	32	43.3	−2.1	0.68
		Females	CTRL	16	54.3		
			DRUG	16	54.4	0.1	0.99
		Males	CTRL	16	36.6		
			DRUG	16	32.3	−4.3	0.37
	Peak to Death	All	CTRL	32	34.6		
			DRUG	32	34.9	0.4	0.91
		Females	CTRL	16	32.9		
			DRUG	16	29.4	−3.4	0.44
		Males	CTRL	16	36.3		
			DRUG	16	40.4	4.2	0.38
Body weight change (g)	Maximum	All	CTRL	32	2.6		
			DRUG	32	3.2	0.6	0.12
		Females	CTRL	16	3.2		
			DRUG	16	3.7	0.5	0.35
		Males	CTRL	16	2.1		
			DRUG	16	2.7	0.6	0.15
	Median	All	CTRL	32	1.6		
			DRUG	32	2.1	0.6	0.07
		Females	CTRL	16	2.0		
			DRUG	16	2.6	0.6	0.21
		Males	CTRL	16	1.1		
			DRUG	16	1.7	0.6	0.14

1 Daily body weight for each individual animal from study start until death was evaluated and treatment group mean values and statistical probabilities from Student's 2-tailed independent t-test are presented. The difference between DRUG and CTRL groups (D-C) is also given.

2 Peak change from initial body weight was detected after spline smoothing (stiffness 100) of each animal's body weight change over time during the period from study start to death.

#### Neuromotor Disease Progression

B6-SJL-SOD1^G93A^/Gur1 mice develop a disease phenotype with severely affected hind-limbs. The first obvious disease symptoms are abnormal hind-limb splay reflexes that are revealed when the mice are suspended by their tails. The symptoms progress to hind-limb digit paresis. Eventually, the mice can no longer use their hind limbs for forward locomotion before succumbing to a more generalized weakness. The weakness progresses until the animals are unable to right themselves when placed on their sides. In this study we employed our B6-SJL-SOD1^G93A^/Gur1 mouse neuromotor scoring system to track the disease progression as previously described. [26]

**Figure 3:**
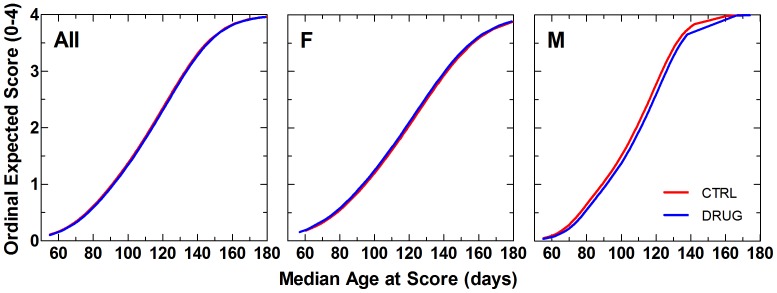
Ordinal logistic regression of neurological severity score vs. median age at score in SOD1^G93A^ mice. Left panel shows data from all animals (All); middle and right panels show data from females (F) and males (M), respectively. Vehicle control (CTRL) animals received 0.22 micron filtered animal facility drinking water. Dexpramipexole-treated (DRUG) animals received daily 200 mg/kg/day dexpramipexole (1.19 g/L) in filtered drinking water. Scores advance in severity from 0 to 4. Results of statistical analysis for these data are given in Table 3.

The daily average neurological disease severity score for the treatment groups was plotted over 180 days (study termination) (Fig. 2, bottom panels). To conserve the mean values as animals began to die, the last values were carried forward during computation of the means. Drug group animals showed a slightly less rapid progression in neurological disease severity score during the early onset phase of the disease by ordinal regression analysis (Fig. 3). This effect was more apparent in males than females.

Because neurological disease severity was assessed using ordinal scores (0–4), the rate of symptomatic neurological disease progression in control and RPPX-treated animals was compared using an ordinal logistic regression of the neurological disease severity score by median age at score. The overall rate of symptomatic disease progression was similar in both groups. Males appeared to be right-shifted (delayed disease progression) by about three days. However, these differences were not statistically significant (p = 0.31, Table 3).

**Table 3:** Effect of Treatment on Neurological Severity Score.^1^

Effect	Test	Subjects	ChiSq	Prob > ChiSq
Treatment	Likelihood-ratio	All	0.099	0.75
		Female	0.075	0.78
		Male	1.042	0.31
	Wald	All	0.095	0.76
		Female	0.073	0.79
		Male	1.009	0.32

Daily neurological severity scores were taken from study start until death. These ordinal scores ranging from 0 to 4 were modeled in relation to the animal's median age at each score level using ordinal logistic regression. The model fits cumulative response probabilities to the logistic function of a linear model using maximum likelihood. Likelihood-ratio and Wald Chi-square test probabilities are provided for the treatment effect. Plots comparing fitted neurological score over time by treatment are shown in Fig. 3.

#### Paralysis Onset and Survival

**Figure 4:**
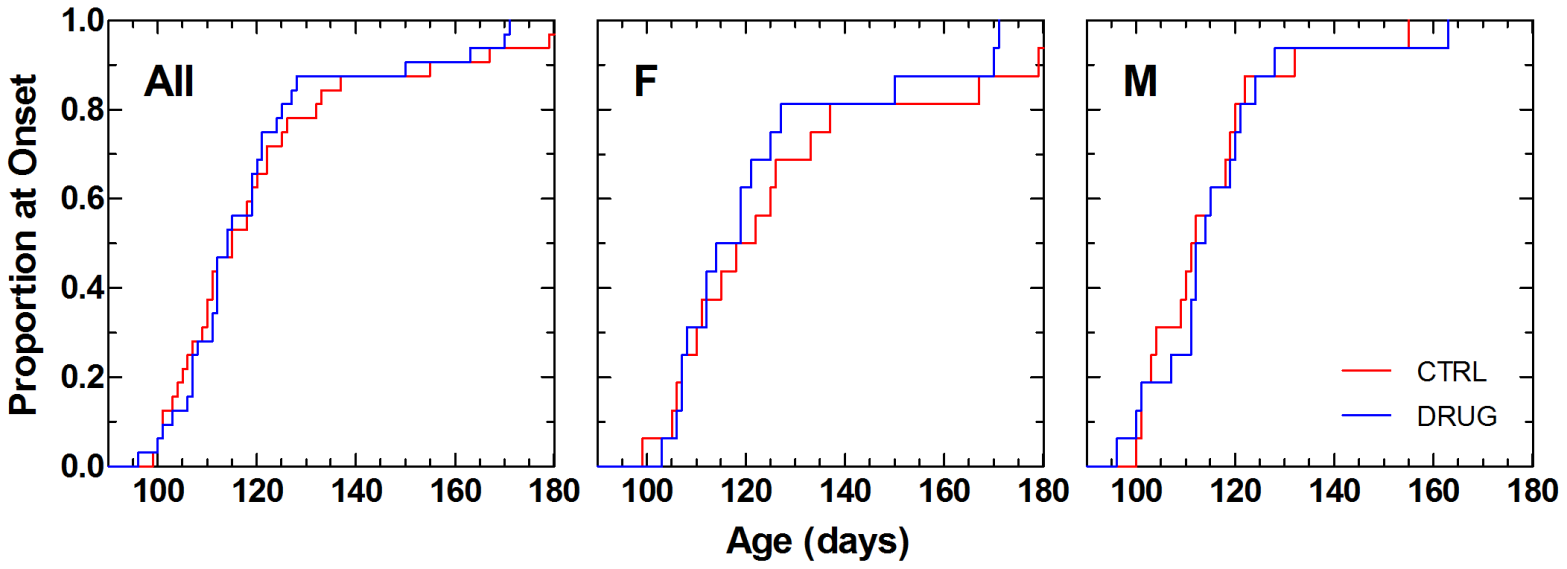
Kaplan-Meier time-to-failure plot for onset of symptomatic neurological disease in SOD1^G93A^ mice. The age at which mice attain a neurological severity score of 2 is taken to be definitive onset of symptomatic disease. Left panel shows data from all animals (All); middle and right panels show data from females (F) and males (M), respectively. Vehicle control (CTRL) animals received 0.22 micron filtered animal facility drinking water. Dexpramipexole-treated (DRUG) animals received daily 200 mg/kg/day dexpramipexole (1.19 g/L) in filtered drinking water. Results of statistical analysis for these data are given in the upper portion of Table 4.

The proportion of mice that progressed to definitive onset of paralytic disease over time is shown in Fig. 4. Overall, there was no statistically significant difference in onset of disease (Table 4).

**Table 4:** Time-to-Event Analysis for Onset of Neurological Symptoms and for Survival Duration.^1–3^

Age (days)	Subjects	Trtmnt	Failed	Censored	Median Time	Test	Prob > ChiSq
Onset	All	CTRL	31	1	115.0	Log-Rank	0.66
		DRUG	32	0	114.0	Wilcoxon	0.96
						Likelihood	0.70
	Female	CTRL	15	1	120.0	Log-Rank	0.47
		DRUG	16	0	116.5	Wilcoxon	0.71
						Likelihood	0.56
	Male	CTRL	16	0	111.5	Log-Rank	0.67
		DRUG	16	0	113.0	Wilcoxon	0.68
						Likelihood	0.61
Death	All	CTRL	29	3	129.5	Log-Rank	0.55
		DRUG	31	1	126.0	Wilcoxon	0.80
						Likelihood	0.43
	Female	CTRL	13	3	137.5	Log-Rank	0.43
		DRUG	15	1	130.0	Wilcoxon	0.56
						Likelihood	0.39
	Male	CTRL	16	0	124.0	Log-Rank	0.92
		DRUG	16	0	124.5	Wilcoxon	1.00
						Likelihood	0.93

Testing Terms: In Kaplan-Meier analysis the Log-Rank test places more weight on later event times; the Wilcoxon test places more weight on early event times and is the optimum rank test if the error distribution is logistic. Prob > ChiSq lists the probability of obtaining, by chance alone, a Chi-square value greater than the one computed if the time-to-event functions are the same for all groups. In Cox proportional hazards analysis the Effect Likelihood Test is the likelihood-ratio Chi-square test on the null hypothesis that the parameter estimate for the Treatment covariate is zero (no effect of treatment). Testing term descriptions are taken from the JMP 10.0.0 Help file. A neurological score of two in both hind limbs is taken to be the definitive onset of neurological symptoms. Animals that did not reach a neurological severity score of 2 prior to termination of the experiment at 180 days of age were right-censored in the onset analysis. Animals that did not die by 180 days of age were right-censored in the survival analysis.

**Figure 5:**
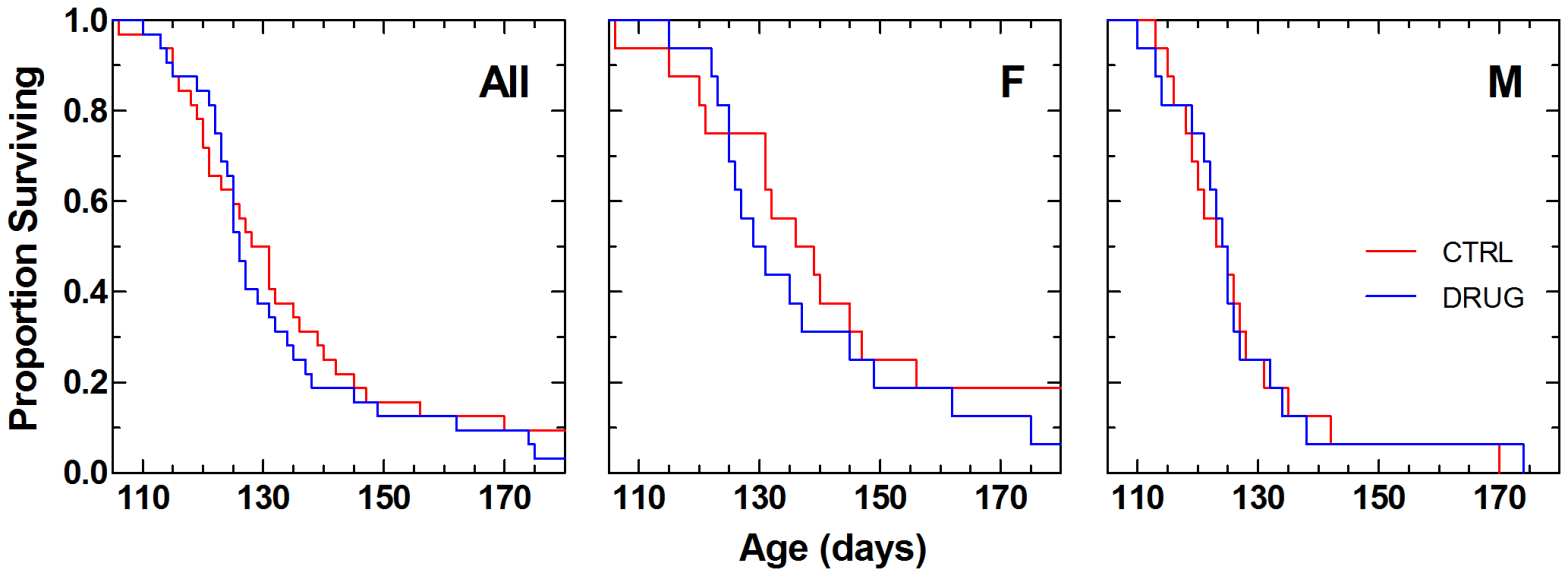
Kaplan-Meier survival plot for age at death in SOD1^G93A^ mice. Left panel shows data from all animals (All); middle and right panels show data from females (F) and males (M), respectively. Vehicle control (CTRL) animals received 0.22 micron filtered animal facility drinking water. Dexpramipexole-treated (DRUG) animals received daily 200 mg/kg/day dexpramipexole (1.19 g/L) in filtered drinking water. Results of statistical analysis for these data are given in the lower portion of Table 4.

The proportions of mice surviving over time are shown in Fig. 5. There was no statistically significant difference in survival proportions over time when comparing control with RPPX-treated high-copy SOD1^G93A^ mice (Table 4).

### *In Vitro* TDP43 Rat Primary Cortical Neuron High-Content Survival Assay

We next wanted to determine if RPPX mitigated toxicity in our primary neuron model of TDP43-mediated cell death and antecedent pathology. Cortical neurons were transfected at 4 days *in vitro* (DIV) with either EGFP, TDP43-EGFP, TDP43^A315T^-EGFP, or TDP43^M337V^-EGFP. Cells were simultaneously transfected with mApple to control for transfection efficiency and normalization of transgene expression. Neurons were imaged 24 hours after transfection and every 24 hours thereafter for 7 days. As we showed previously, over-expression of wild-type TDP43-EGFP or TDP43-EGFP bearing mutations associated with familial ALS, significantly increased the risk for neuronal death over EGFP alone (Table 5).

**Table 5:** Cortical Neuron Survival Data, No RPPX Treatment.

	n	Events	HR	95% CI	p
EGFP	1361	986	1	—	—
TDP43-EGFP	770	690	1.7592	1.591–1.945	2×10^−16^
TDP43^A315T^-EGFP	668	584	1.7729	1.595–1.97	2×10^−16^
TDP43^M337V^-EGFP	1155	1032	1.6998	1.556–1.856	2×10^−16^

**Figure 6:**
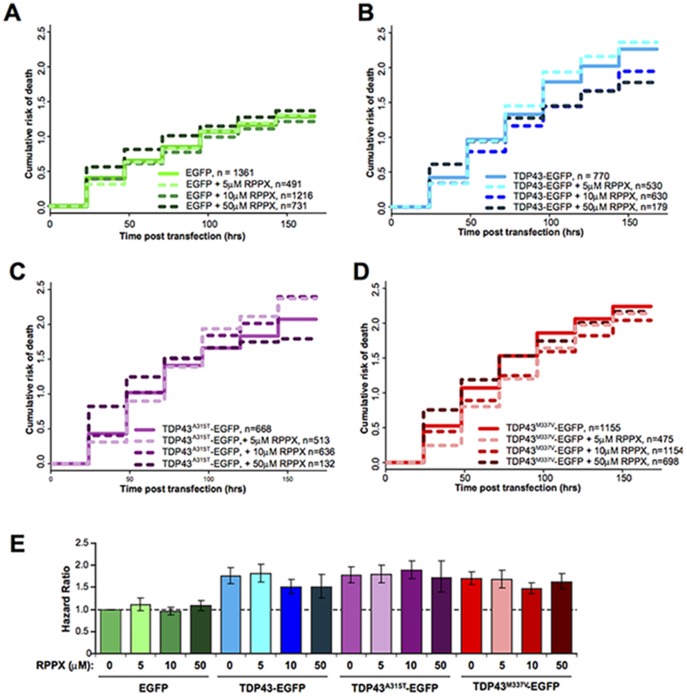
RRPX has little effect on neuronal survival in TDP43 overexpression model of ALS. (A–D) Kaplan-Meier cumulative hazard plots for neurons treated with RPPX at doses indicated for neurons transfected with mApple and (A) EGFP, (B) wildtype TDP43-EGFP, (C) TDP43^A315T^-EGFP, (D) TDP43^M337V^-EGFP. (E) Hazard ratios relative to control (EGFP, no RPPX) for all conditions shown above. Data for each condition are pooled from n = 2–5 biological replicates.

Previous work has indicated that concentrations of RPPX between 1 and 100 µM may improve mitochondrial function and improve cell viability under conditions of oxidative stress. [15] We tested the effect of RPPX at 5–50 µM (Tables 6–9, Fig. 6). Overall, the treatment had little effect on neuronal survival. In control (EGFP expressing) cells, a slight worsening of survival was observed in the presence of 5 µM RPPX (HR = 1.16, 95% CI = 1.0153–1.33, p = 0.0293), although no such change was observed at higher doses of RPPX (Table 5, Fig. 6). RPPX also had little effect on cell survival in TDP43-expressing neurons (Table 6–8, Fig. 6). A slight improvement was detected both wild-type TDP43-expressing cells, but only at 10 µM RPPX (HR = 0.8543, 95% CI = 0.763–0.956, p = 0.00621) and the M337V mutant (HR = 0.8591, 95% CI = 0.797–0.948, p = 0.00158).

**Table 6:** Cortical Neuron Survival Data, EGFP with RPPX Treatment.

	n	Events	HR	95% CI	p
EGFP	1361	986	1	—	—
TDP43-EGFP	770	690	1.7592	1.591–1.945	2×10^−16^
TDP43^A315T^-EGFP	668	584	1.7729	1.595–1.97	2×10^−16^
TDP43^M337V^-EGFP	1155	1032	1.6998	1.556–1.856	2×10^−16^

**Table 7:** Cortical Neuron Survival Data, TDP43-EGFP with RPPX Treatment.

	n	Events	HR	95% CI	p
EGFP	1361	986	1	—	—
TDP43-EGFP	770	690	1.7592	1.591–1.945	2×10^−16^
TDP43^A315T^-EGFP	668	584	1.7729	1.595–1.97	2×10^−16^
TDP43^M337V^-EGFP	1155	1032	1.6998	1.556–1.856	2×10^−16^

**Table 8:** Cortical Neuron Survival Data, TDP43^A315T^-EGFP with RPPX Treatment.

	n	Events	HR	95% CI	p
EGFP	1361	986	1	—	—
TDP43-EGFP	770	690	1.7592	1.591–1.945	2×10^−16^
TDP43^A315T^-EGFP	668	584	1.7729	1.595–1.97	2×10^−16^
TDP43^M337V^-EGFP	1155	1032	1.6998	1.556–1.856	2×10^−16^

**Table 9:** Cortical Neuron Survival Data, TDP43^M337V^-EGFP with RPPX Treatment.

	n	Events	HR	95% CI	p
TDP43^M337V^-EGFP	1155	1032	1	—	—
TDP43^M337V^-EGFP +5 µ M RPPX	475	419	1.0489	0.922–1.193	0.46723
TDP43^M337V^-EGFP +10 µ M RPPX	1154	1004	0.8691	0.797–0.948	0.00158*
TDP43^M337V^-EGFP +50 µ M RPPX	698	618	0.9227	0.831–1.024	0.13149

## Discussion

In this study, we tested RPPX in two parallel screening systems: 1) appropriately powered, sibling-matched, gender-balanced survival efficacy screening in high-copy B6-SJL-SOD1^G93A^/Gur1 mice, and 2) high-content neuronal survival screening in primary rat cortical neurons transfected with wild-type human TDP43 or mutant human TDP43. In both cases, we sought to expose the test systems to drug levels approximating those achieved and reported in human Phase II clinical investigations. [26] Unfortunately, we found no compelling difference between the control and experimental conditions. Clinically, RPPX has been more extensively studied as a therapeutic intervention to ameliorate ALS disease progression than any other drug, with the notable exception of the FDA-approved drug riluzole. Most recently, a Biogen Idec-sponsored Phase 3 trial titled Empower investigating dexpramipexole in people with ALS was completed. [16] The randomized, double-blind, placebo controlled trial enrolled 943 people with ALS, the largest ALS clinical trial to date.

Complete results from an earlier two-part Phase 2 trial investigating RPPX in people with ALS were published in 2011. These results indicated a significant difference between groups in a joint rank test of change from baseline using ALS Functional Rating Scale scores (ALSFRS-R) and mortality. [23] A very similar joint rank analysis was used in the Empower trial above and was referred to as combined assessment of function and survival (CAFS), where no differences were observed between treatment groups and placebo control. [16] The disparity in results between the two trials has not yet been completely explored, but may be attributable to a combination of factors. For example, the smaller Phase 2 trial may not have been adequately powered to avoid type I error with a joint rank analysis of variables that are not completely independent (i.e., ALSFRS-R and mortality). Second, the smaller Phase 2 trial may have inadvertently been enriched with patients who responded well to dexpramipexole. Unfortunately, without a predictive clinical biomarker for dexpramipexole efficacy in ALS patients, the latter explanation, while optimistic, does not provide a means for patient stratification during clinical trial assignment.

The results from our studies more closely align with the results from the large Phase 3 clinical trial described above. Compelling signs of efficacy and neuroprotection by RPPX were not observed in either of our ALS drug screening systems. These systems reflect both mutant SOD1- and TDP43-mediated forms of neurodegeneration. The systems also reflect both complex non-cell autonomous and neuronal cell autonomous disease mechanisms.

In SOD1^G93A^ mice, no effect was observed on neuromotor disease progression or survival. RPPX-treated mice appeared to maintain body weight better than vehicle-treated mice, but the effect was not statistically significant. Our results obliquely contrast with those published by Danzeisen et al in 2006 where efficacy by RPPX was observed B6-SJL-SOD1^G93A^/Gur1 mice. The studies are not directly comparable on multiple counts. In the Danzeisen study, treatment cohorts were not gender balanced and were underpowered according to current internationally accepted guidelines for preclinical studies in B6-SJL-SOD1^G93A^/Gur1 mice. Even more importantly, the doses of RPPX tested in the two studies are different. In the current studies, we tested a RPPX daily exposure of approximately 200 mg/kg in order to test roughly equivalent plasma exposure as that of the recent human clinical trials. In the Danzeisen study, 3 mg/kg/day was tested for efficacy. It is unclear why this lower dose was chosen in the Danzeisen study since they reported results from a drug levels study using a dose of 200 mg/kg/day where observed drug levels in both plasma and brain were measured in the low µM range. All of their reported positive *in vivo* and *in vitro* drug activity results were produced by from dose levels or concentrations much higher than 3 mg/kg/day or 1–6 µM, respectively. CNS-to-Plasma RPPX drug level ratio represents another indirect disparity between the report by Danzeisen et al and ours. In the former, the brain:plasma drug level was approximately 5∶1. In the latter, we looked instead at spinal cord:plasma ratios and found it to be approximately 1∶1. We typically choose to study spinal cord levels in B6-SJL-SOD1^G93A^/Gur1 related pharmacology studies because it is a site of primary pathology in the model better characterized by us. While spinal cord and brain drug levels are typically similar, it is possible that the differences in reported CNS-to-plasma drug ratios could be explained by the different tissues studied.

In primary cortical neurons transfected with either mutant or wild-type human TDP43, a marginally significant improvement in a single indicator of neuronal survival was observed, and only at the 10 µM RPPX treatment.

The results of these experiments, taken in context with results produced by other molecules tested in both screening systems, do not argue positively for further study of RPPX in ALS. [6], [11], [12], [26], [27]

## Methods

### Dexpramipexole Analytical Chemistry

#### Sample and Dexpramipexole Standards Preparation

Dexpramipexole dihydrochloride for all experiments herein was purchased from Toronto Research Chemicals (Catalogue #: P700745). For analytical chemistry standard preparations, five microliter aliquots of standards were prepared in 1∶1water:acetonitrile and added to 45 µL of blank control plasma or blank spinal cord homogenate into 1 mL 96 deep well plate. One hundred fifty microliters of acetonitrile containing 500 ng/mL analytical internal standard (metformin) and 0.1% formic acid were added to the each well. The plate was vortexed then centrifuged for 30 minute at 3000 rpm at 4°C. One hundred microliters of the supernatant were pipetted into a new 96 well plate for LC-MS/MS analysis. Plasma and spinal cord homogenate samples from mice treated with RPPX were handled similarly without RPPX spiking steps. Quantitation of RPPX in samples from treated mice was performed against a calibration curve generated by spiking the analyte into the appropriate matrix (10, 100, 200, 500, 1000, 2000, 5000 and 10000 ng/mL final concentrations).

#### High Performance Liquid Chromatagraphy (HPLC) Conditions

HPLC was performed using Shimadzu LC-10AD vp pumps and SIL-HTc autosampler with a Phenomenex luna CN, 50×2.1 i.d. 4 4 µm column. The buffer solution used was 0.1% formic acid in water. The aqueous reservoir was loaded with 0.1% formic acid in 10 mM ammonium formate. The organic reservoir was loaded with 0.1% formic acid in acetonitrile. Injection volume for plasma and spinal cord homogenates was 10 µL. The run time was 6.0 mins at ambient temperature.

#### Mass Spectrometer Conditions

The instrument used was PE Sciex API3000 with an Electrospray interface set to multiple reaction monitoring model with CUR11, NEB11, and CAD10 gases and set at 450°C.

### Dexpramipexole Survival Efficacy Study

We used the rigorous survival study methods described in detail by Scott, et al. [9] summarized briefly below, for the current study. These study methods are derived from statistical analyses of thousands of untreated B6-SJL-SOD1^G93A^/Gur1 mice and reduce variability and thereby reduce the number of mice necessary to reliably detect a statistically significant improvement in survival.

#### Animals

This study was approved by the ALSTDI Institutional Animal Care and Use Committee (IACUC) and in accordance with the Institute for Laboratory Animal Research (ILAR) Guide for Care and Use of Laboratory Animals [18]. Mouse cages were each supplied with autoclavable red mouse igloos to diffuse light and increase mouse sense of security (Bioserve, Maryland Product #K337). In all animal studies described herein, mice were singly housed under specific pathogen free conditions. Food and water were provided ad libitum. The diet used was Teklad Global diet #2918 for rodents (Harland Laboratories, Houston, TX). Drinking water was refreshed twice weekly in graduated water bottles. Amount of water consumed was estimated by measuring the difference between the volume of water on the day when it was refreshed and the volume remaining prior to the next refilling. Amount of water consumed was recorded and compared between RPPX treated mice and vehicle control treated mice to determine whether RPPX in drinking water resulted in taste aversion (S1 Database). No differences were observed (data not shown). RPPX loaded drinking water was prepared by adding 1.505 grams of dexpramipexole dihydrochloride to 1.260 L drinking water. RPPX loaded drinking water and normal vehicle drinking water were prepared freshly in the morning twice weekly. Drinking water was the preferred route of administration for RPPX because RPPX has known oral bioavailability. We verified this in the current studies (Fig. 1). Further, ad libitum drinking water drug administration is non-invasive and did not cause taste aversion or dehydration.

High-copy SOD1G93A transgenic mice bred under contract for ALS TDI by BRM/Biomere, LLC. were used for this study. This mouse colony was derived from the B6SJL- TgN(SOD1G93A)1Gur strain, originally obtained from The Jackson Laboratory (Bar Harbor, Maine) and initially designed and generated by Gurney et al. [12]. BRM maintains the colony by crossing B6-SJL sires harboring the transgene with non-transgenic B6-SJL dams. Transgenic mice are shipped to ALS TDI at 35–45 days of age. Mice are allowed at least one week to acclimate to ALS TDI's animal facility (a 12-h light/dark cycle) before being assigned to a study. Copy number is determined by qPCR analysis on Taqman 7900 of genomic DNA derived from ear punch samples from each mouse. Animals were monitored for neurological disease progression according to the protocol previously reported. [26] Neurological scoring procedures and body weight measurements were completed on a bench-top in the animal holding room. All neurological score and body weight data were captured by the custom ALSTDI Laboratory Information Management System (LIMS) (Database S2). End-stage mice were euthanized in a separate procedure room. Euthanasia for animals in drug levels studies and the survival efficacy study was carried out using CO2 by using 100% CO2 at a flow rate of approximately 20% of chamber volume per minute. For the survival efficacy study, animals were euthanized by CO2 when they reached ALS related end-stage defined by an inability of the mouse to right itself within 10 seconds when placed on its side. The observing technician is required to test the animal by placing the animal on both sides. Failure to right itself from either side triggers euthanasia.

#### Experimental Groups

At 50 days of age mice were separated into Drug Treatment (DRUG) and Vehicle Control (CTRL) groups. Prior to any treatment, groups were constituted so as to minimize between- group variability by using the following criteria. Groups were balanced with respect to gender (16 males, 16 females per group) and body weight within gender (mean starting weights were typically within 0.3 g for either gender between groups). In addition, groups were age-matched and littermate-matched. Each male and female in the Drug Treatment group had a corresponding male and female littermate in the Vehicle Control group. The study was observer-blinded.

#### Statistical Analysis

Statistical analyses were applied to the B6-SJL-SOD1^G93A^/Gur1 efficacy trial as previously reported. [26]

Briefly, body weight data analyses utilized relevant summary measures as recommended by Matthews, et al. [26], [28] Summary measures derived from body weight change over time included time to attain peak body weight and time from peak body weight to death. These summary measures were detected from spline-fitted changes in body weight over time for each individual animal. These time-to-event measures were then analyzed using Kaplan-Meier survival fit analysis with the Log-Rank and Wilcoxon tests for statistical significance. Cox proportional hazards analysis was also performed to determine hazard ratios and test for statistical significance of their differences using the Effect Likelihood Chi Square test.

A summary measure derived from daily ordinal neurological severity scores counted days at score for each animal at each score level. These data were analyzed using categorical analysis where Chi Square tests were used to determine whether any differences in score frequencies were statistically significant. Degrees of freedom were based on the number of animals in each group rather than total number of scores.

Time to onset of definitive disease and survival time were also analyzed using the Kaplan-Meier and Cox proportional hazard methods. Statistical analyses were performed using JMPH 7.0.1, SAS Institute, Inc., SAS Campus Drive, Cary, NC 27513, USA. Cox proportional hazard fitting, using litter as a frailty term, was performed using StataH/IC 10.1, 4905 Lakeway Drive, College Station, TX 77845, USA. P-values less than 0.05 were taken to be statistically significant.

### *In Vitro* TDP43 Rat Primary Cortical Neuron High-Content Survival Assay

#### Plasmids

Human TDP43 was cloned into pGW1-CMV plasmid, C terminally fused to EGFP as previously described. [11] The M337V mutation was created by site-directed mutagenesis (Stratagene). EGFP and mApple were cloned into pGW1-CMV as previously described. [11]

#### Cell culture

Cortical neurons were isolated from E20–21 Long Evans rat embryos (Charles River) and cultured at 100,000 cells/well of 96 well plate in serum-free Neurobasal medium (Invitrogen) supplemented with B27, GlutaMax, and pen/strep (Invitrogen). At 4 days *in vitro* (DIV), neurons were transfected with Lipofectamine2000 (Invitrogen) as per the manufacturers protocol. Neurons were cotransfected with plasmids encoding survival markers (mApple) or proteins of interest (TDP43-EGFP or EGFP control) in a 1∶1 molar ratio for a total of 0.2 µg total DNA per well. After transfection cells were returned to Neurobasal media mixed 1∶1 with conditioned media. For cells treated with dexpramipexole, drug was added once, immediately after transfection.

#### Robotic microscopy

For neuronal survival analysis, we use a robotic imaging system described by Arrasate and Finkbeiner. [11]–[13] Briefly, images were obtained with an inverted Nikon microscope (Ti-E) equipped with PerfectFocus, an extra-long working distance (ELWD) 20x objective lens, and a back-illuminated Andor iXON 888 14-bit, cooled, electron multiplying charge coupled device (EMCCD). Illumination was provided a xenon lamp and liquid light guide. All movements of the stage are controlled with electrical stepper motors. Coordination of fluorescence excitation and emission filters, stage movements, focusing, and imaging acquisition was accomplished with custom-designed and commercially available programs.

#### Image analysis and statistics

Digitized images were assembled into montages in Pipeline Pilot and ImageJ using original programs. Background fluorescence from neighboring regions of interest was subtracted, montages from each time point are assembled into stacks in chronological order and aligned with one another. Cell bodies of transfected neurons, identified by morphology marker fluorescence (mApple), were automatically segmented and followed over time. Cell death was determined by an abrupt loss of fluorescence, indicating shrinkage or disappearance of the cell body. The time of death for each neuron was considered the last time that the neuron was present. Kaplan-Meier and cumulative risk of death curves were generated in R. Statistical significance of survival differences between cohorts of neurons is determined by the log-rank test, and Cox proportional hazards analysis used to measure the relative change in the risk of death attributed to various experimental conditions.

## Supporting Information

S1 Fig **Mass spectrophotometry data demonstrating stable formulation of dexapramipexole in drinking water.**
(PDF)

S1 Database **A database of all neurological score and body weight data for all mice included in the survival efficacy study.**
(XLSX)

S2 Database **A database of all drinking water consumed by mice in the survival efficacy study.**
(XLS)
