# Supplementary material for: Dexpramipexole Is Ineffective in Two Models of ALS Related Neurodegeneration
Source: PLoS One. 2014 Dec 19;9(12):e91608. doi: 10.1371/journal.pone.0091608 (PMC4272269; doi:10.1371/journal.pone.0091608)

Supplemental Figure 1a

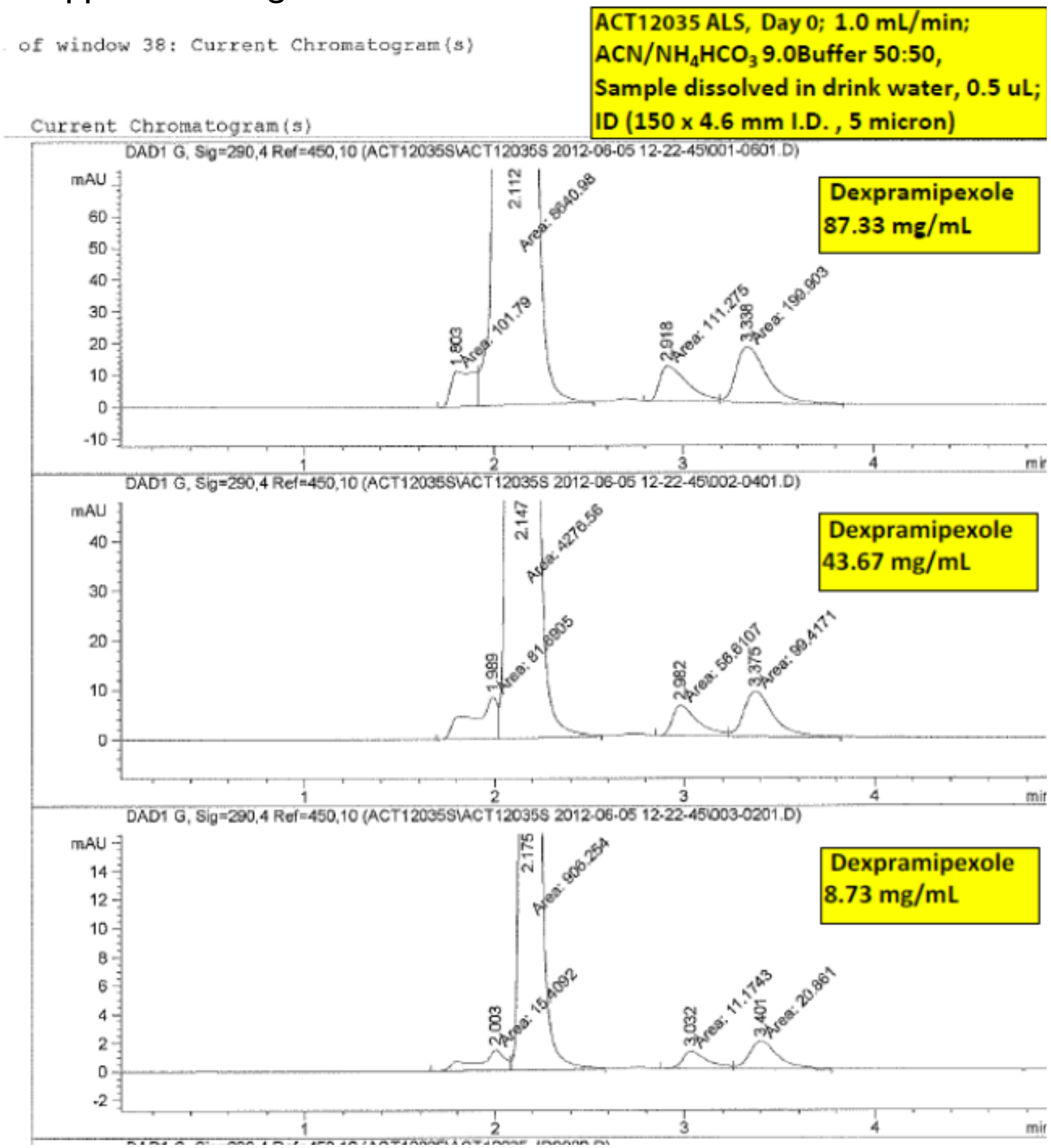

Supplemental Figure 1b

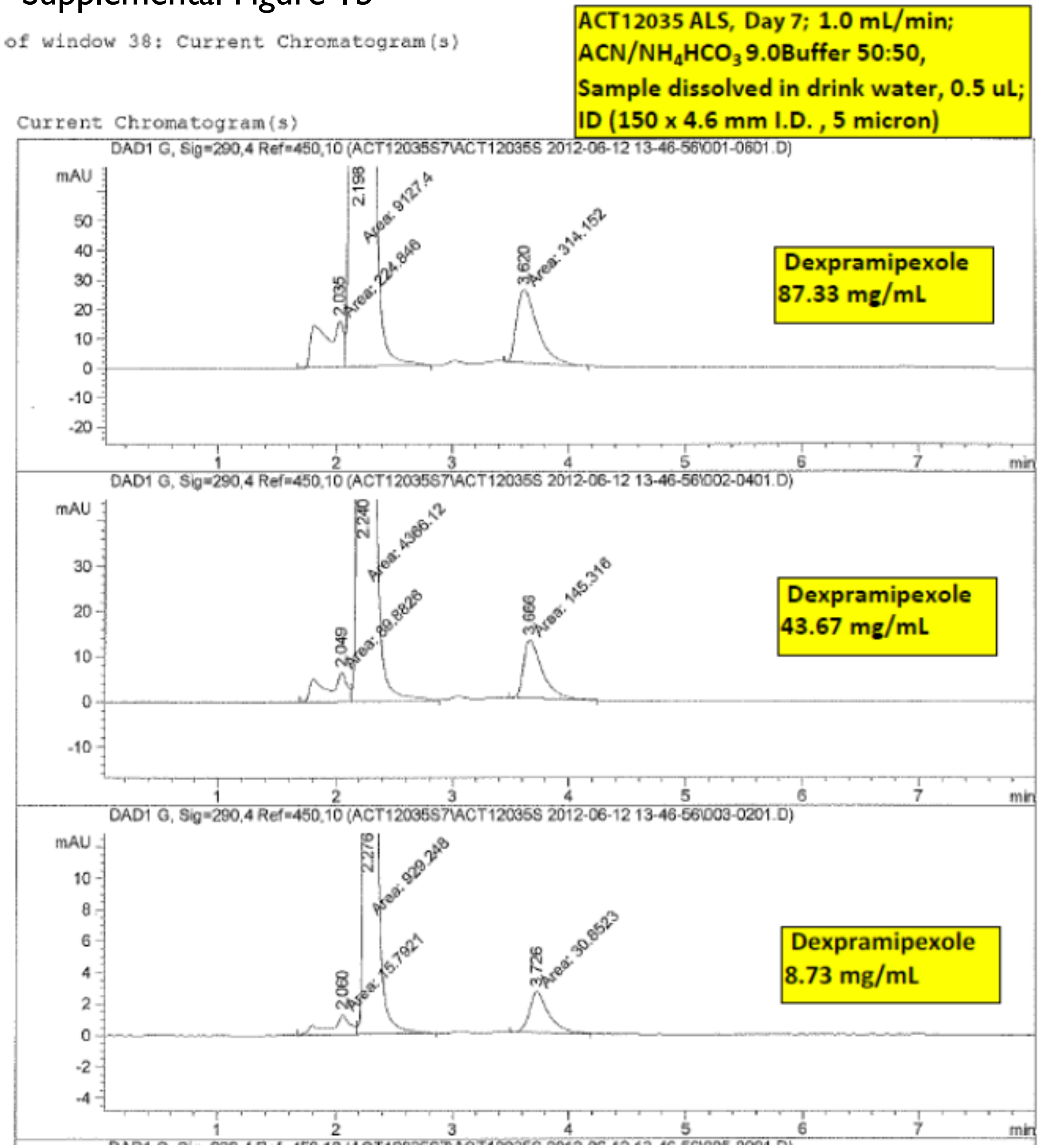

Supplement: S1 Fig — Mass spectrophotometry data demonstrating stable formulation of dexapramipexole in drinking water. (PDF) [file pone.0091608.s001.pdf]
